# Implementation Outcomes of an Intervention to Improve Myocardial Infarction Care in Tanzania

**DOI:** 10.5334/aogh.4651

**Published:** 2025-08-05

**Authors:** Julian T. Hertz, Francis M. Sakita, Zaheer Rik Munshi, Faraan O. Rahim, Daniel Mganga, Arafa Kachenje, James J. Munisi, Abigail S. Pyne, Prosper Bashaka, Adamu Kilungu, Ayshat Mussa Aboud, Hayden B. Bosworth, Janet Prvu Bettger

**Affiliations:** 1 Department of Emergency Medicine, Duke University, Durham, North Carolina, USA; 2 Duke Global Health Institute, Durham, North Carolina, USA; 3 Kilimanjaro Christian Medical Centre, Moshi, Tanzania; 4 Kilimanjaro Christian Medical University College, Moshi, Tanzania; 5 The College, University of Chicago, Chicago, Illinois, USA; 6 Harvard Medical School, Harvard University, Cambridge, Massachusetts, USA; 7 Kibosho Hospital, Kibosho, Tanzania; 8 Tanga Regional Hospital, Tanga, Tanzania; 9 Mbeya Zonal Referral Hospital, Mbeya, Tanzania; 10 Mnazi Mmoja Hospital, Zanzibar, Tanzania; 11 Department of Population Health Sciences, Duke University, Durham, North Carolina, USA; 12 Department of Physical Medicine & Rehabilitation, University of North Carolina School of Medicine, North Carolina, USA

**Keywords:** myocardial infarction, fidelity, penetration, implementation science, Sub-Saharan Africa, emergency department

## Abstract

*Background:* Uptake of evidence-based care for acute myocardial infarction (AMI) is limited in Tanzania. To address this, a tailored intervention, the Multicomponent Intervention to Improve Acute Myocardial Infarction Care (MIMIC), was co-designed by an interdisciplinary team.

*Objectives:* To determine implementation outcomes from a pilot trial of the MIMIC intervention in a Tanzanian emergency department (ED).

*Methods:* The MIMIC intervention was implemented by the ED staff for one year. Fidelity, penetration, and costs were observed for each of the intervention components: designated champions to audit care, an online training module for staff, a triage card for nurses to flag patients with AMI symptoms, pocket cards summarizing AMI management for physicians, and an educational pamphlet for patients. Thirty days following enrollment, patient participants were contacted via telephone to inquire whether they had read the pamphlet.

*Results:* Physician champions and nurse champions were actively engaged in the intervention across the twelve-month study period. Fidelity to the pocket card was excellent, with all 22 (100%) physicians observed to have ever brought their pocket cards to work, and penetration across physician-shifts was 96.1% (1835/1910). The training module was started by 20 out of the 22 (91%) physicians and 25 of the 32 (78%) nurses observed. Penetration, measured by module completion, was the same for physicians (20 of 22, 91%) but lower among nurses (21 of 32, 65.6%). Triage cards were used for 453 out of the 577 (78.5%) patients with chest pain or dyspnea. Fidelity to patients with AMI receiving the educational pamphlet was 37.6% (53 of 141). Only 22 of the 39 (56%) surviving AMI patients who received the pamphlet reported reading it, with most of the rest reporting being unaware that they had received it. The total annual cost of the MIMIC intervention was USD 1324.24.

*Conclusions:* There was high variability in fidelity and penetration of the individual intervention components. Future studies should explore reasons for incomplete penetration and analyze cost-effectiveness for scale-up efforts across Tanzania.

## Introduction

As Sub-Saharan Africa (SSA) proceeds through the epidemiologic transition, the burden of life-threatening cardiovascular emergencies such as acute myocardial infarction (AMI) is growing rapidly [1–3]. Current studies estimate that AMI is responsible for hundreds of thousands of deaths annually in SSA [4]. In Tanzania, a recent modeling analysis found that the local incidence of AMI-related mortality exceeds that of the United States and Europe [3, 5]. This rise in cardiovascular mortality has created new challenges for health systems in SSA, which were historically focused on communicable diseases [6]. In Tanzania, a combination of contextual factors, including limited provider training, inadequate patient knowledge, insufficient resources, and other barriers to care have led to poor uptake of evidence-based AMI care and poor outcomes [7, 8]: observational studies have found that approximately 90% of AMI cases are misdiagnosed, that patients with AMI rarely receive evidence-based therapies such as aspirin, and that over 40% of patients with AMI die within 30 days of hospital presentation [5, 9, 10].

Improving the uptake of evidence-based care for AMI in Tanzania requires an approach rooted in implementation science, as the benefits of such care are clear [11, 12], but substantial gaps in actual clinical practice persist. Thus, an interdisciplinary team of implementation scientists, physicians, nurses, and social scientists partnered with key local stakeholders to co-design a quality improvement intervention: the Multicomponent Intervention to Improve Acute Myocardial Infarction Care in Tanzania (MIMIC) [13, 14]. This intervention, which was developed using implementation mapping to identify targeted strategies to overcome contextual barriers [14], consists of five key components: champions, provider training, pocket cards, triage reminder cards, and patient education [13].

MIMIC is the first published quality improvement intervention for AMI care in SSA, but its implementation outcomes and effectiveness are unknown. In this study, we report implementation outcomes from a pilot trial of MIMIC in the emergency department (ED) of Kilimanjaro Christian Medical Centre (KCMC) in northern Tanzania. Determining the fidelity, cost, and penetration of MIMIC in this trial will provide context for examining effectiveness and inform future efforts to scale up interventions like MIMIC across Tanzania and SSA.

## Methods

### Study setting

The observational study was conducted at Kilimanjaro Christian Medical Centre (KCMC), a regional referral hospital in northern Tanzania. No referral is necessary for presentation to the KCMC ED, which serves the local population of Moshi as well as a broader catchment area of 15 million people. Previous studies in the KCMC ED demonstrated suboptimal use of evidence-based AMI care, including low rates of diagnostic testing and evidence-based treatment [5, 9, 10, 15]. KCMC is equipped with troponin assays, electrocardiography (ECG) machines, and AMI medications including thrombolytics, anti-platelets, anticoagulants, and blood pressure medications. At the time of this study, KCMC did not have the capacity for coronary angiography, percutaneous coronary intervention, or cardiac surgery. Because KCMC is a teaching hospital, multiple trainees rotate through the ED, including emergency medicine residents, internal medicine residents, interns, nurse interns, and medical students.

### MIMIC intervention and implementation

The MIMIC intervention was designed to address suboptimal identification of patients presenting to the ED with AMI symptoms, use of electrocardiograms or cardiac biomarker testing for diagnosis, treatment using aspirin and other evidence-based therapies, and patient education [5, 9]. Longitudinal evaluations of care, shareholder interviews, implementation mapping, and iterative co-design led to the MIMIC intervention, which includes five specific strategies to improve the uptake of evidence-based AMI care: identification of champions, physician and nurse provider training, pocket cards for physicians, triage reminder cards for nurses, and patient education provided in writing [13, 14]. The first MIMIC component was clinical champions. Two champions (one physician and one nurse) were appointed by the Department Head to audit AMI care, encourage staff, and ensure implementation of the other intervention components. On a monthly basis, champions would audit care for recent AMI patients and give feedback to providers in cases where care was suboptimal. Champions were also responsible for ensuring all staff members completed the online training module and received pocket cards, as described below. In addition to overseeing the implementation of each of these strategies, the champions also recognized providers who provided excellent care with congratulatory certificates. Champions received a supplemental stipend of 100,000 Tanzanian shillings (approximately 40 USD) each per month for their efforts. The second MIMIC component was an online training module. This brief online training module was distributed to all ED physicians and nurses. The module outlined best practices in AMI diagnosis and treatment and reviewed common misconceptions in AMI care. The third intervention component was a unique “AMI suspect” triage card that a triage nurse placed on the stretcher of any adult patient with chest pain or dyspnea. This special triage card was intended to prompt the treating physician to consider the diagnosis of AMI. The fourth intervention component was pocket cards summarizing AMI diagnosis and treatment, which were distributed by the champions to all ED staff. The final intervention component was an educational pamphlet which was given to patients diagnosed with AMI and displayed in the waiting room. Additional details of the intervention, including all intervention materials, are published elsewhere [13]. MIMIC was implemented by KCMC ED staff; the research team was not involved in the day-to-day implementation of the intervention. Implementation of the intervention began on September 1, 2023, and the pilot trial was conducted for one year (from September 1, 2023 through September 1, 2024) [16].

### Patient participants and provider populations

Adult patients (age ≥18 years) presenting to the ED with possible AMI symptoms (chest pain or dyspnea) were approached by research assistants and offered enrollment in the study. After obtaining informed consent, the research staff observed the care of enrolled participants. For study purposes, AMI was defined by any of the following, as per Fourth Universal Guidelines [17]: (1) documented final hospital discharge diagnosis of AMI, (2) ECG demonstrating STEMI, (3) pathologically elevated serum troponin (>99th percentile of manufacturer defined normal range) with repeat 3-hour troponin >11% higher or lower than the initial troponin, or (4) in cases when only a single troponin value was available, a pathologically elevated serum troponin in a participant without advanced renal disease (estimated glomerular filtration rate (GFR) >15 ml/min/1.73 m^2^). For study purposes, all ECGs underwent external review by physician adjudicators. ECGs were read by two independent physicians, residency trained in emergency medicine, to determine the presence of ST-elevation myocardial infarction (STEMI), as per the Fourth Universal Definition of Myocardial Infarction Guidelines [17]. In cases of disagreement, a third physician adjudicator served as a tiebreaker. Research staff collected results of diagnostic testing among participants, including ECGs, serum troponins, and serum creatinine levels, as well as hospital discharge diagnoses directly from the medical record.

Implementation outcomes were assessed for all physicians and nurses working in the KCMC ED. The total number of physicians and nurses working in the KCMC ED during the study period was taken directly from the ED staff log.

### MIMIC implementation evaluation

The full study protocol, including plans for assessing implementation outcomes, has been previously published [16]. On a daily basis, from 8 AM until 11 PM, members of the research team directly observed ED physicians and nurses’ use of AMI pocket cards and the “AMI Suspect” triage cards. Research staff also monitored all costs associated with printing MIMIC materials, including pocket cards, triage cards, educational pamphlets, and congratulatory certificates.

Thirty days following enrollment, patient participants were contacted via telephone to ascertain whether they had read the educational pamphlet. For patients who reported receiving and reading the educational pamphlet, the four-item Acceptability of Intervention Measurement [18] survey was administered to assess the acceptability of the pamphlet to patients. At the conclusion of the one-year pilot trial, the registry of persons starting and completing the online AMI training module was cross-checked with the KCMC ED staff roster to determine the proportion of KCMC ED staff who had started and completed the module, respectively.

Fidelity, the degree to which an intervention was implemented as intended [19], was assessed for each of the MIMIC components. The triage card component was evaluated as the proportion of enrolled patient participants with AMI symptoms who were correctly identified and flagged with “AMI Suspect” cards. Fidelity to pocket card use was assessed as the proportion of ED physicians observed to ever have their pocket cards with them at work. Fidelity to the training module was defined by the proportion of ED staff who started the training module, reported for all ED staff for the year and by provider type (physicians and nurses). Finally, the proportion of patient participants meeting the study definition of AMI who received an educational pamphlet in the ED was used to measure fidelity to the patient’s educational component. A priori fidelity threshold of 80% was considered optimal. In addition to reporting overall fidelity, monthly fidelity to the various intervention components was also tracked. Monthly fidelity to the champion intervention was defined as having both the nurse champion and the physician champion conduct an audit of AMI care at least once in a given month. Monthly fidelity to the other intervention components (the proportion of ED physicians observed to have the pocket card with them at work at least once in a given month, the cumulative proportion of ED staff starting the training module each month, the proportion of patients with chest pain or dyspnea receiving the “ACS Suspect” triage card each month, and the proportion of patients with AMI receiving the educational pamphlet each month) was also tracked prospectively by the study team.

Penetration was assessed as the degree to which the individual MIMIC components were integrated at KCMC as part of routine care. For study purposes, each physician and nurse observed during each shift was treated as a discrete observation. Thus, for the purposes of assessing penetration of triage cards and pocket cards, each shift was counted as a new observation for each physician and nurse. Physicians in the KCMC ED work 12-hour shifts, and typically three physicians are assigned to each shift, resulting in 1910 discrete physician-shifts observed during this one-year study. Depending on staffing, either two or three nurse triage-shifts occur each day, with one nurse assigned to work in triage during each shift; this resulted in 851 discrete triage nurse-shifts observed during the study. For KCMC ED clinicians, penetration measures included the proportion of triage nurses directly observed to be using the “AMI Suspect” cards during each shift and the proportion of ED physicians observed to have their pocket cards with them at work during each shift. A priori penetration threshold of 80% for provider-specific measures was considered optimal. Additionally, penetration of the online training module was defined by the proportion of all ED providers completing the module. For patients, the penetration measure was the proportion of all participants with AMI surviving to 30 days who reported reading their educational pamphlet.

Financial costs associated with MIMIC implementation, including champion stipends and printing costs for educational pamphlets, pocket cards, triage cards, and congratulatory certificates, were summed and utilized as an additional implementation outcome. To estimate the cost of delivering the online module, the time-driven activity-based costing (TDABC) process was used [20]. To do so, we multiplied the total number of hours a graduate research assistant spent building and revising the online module by their hourly pay.

### Statistical analysis

Statistical analyses were performed in the R Suite. Continuous variables are presented as medians (interquartile ranges) and categorical variables are presented as proportions. Serum creatinine values were converted to estimated GFR using the 2021 CKD-EPI formula [21]. Costs are reported in both Tanzanian shillings (TSh) and US dollars (USD), using the most recent published conversion rate from the World Bank (2,297.76 tsh to 1 USD) [22].

### Research ethics and data availability

Written, informed consent was obtained from all research participants at enrollment upon presentation to the ED. Ethical approval for this study was obtained from institutional review boards at KCMC (Proposal 893, Ver 7, Dec 21st, 2020), Duke Health (Pro00090902, Ver 1.7, Sep 16th, 2020), and the Tanzania National Institute for Medical Research (NIMR/HQ/R.8a/Vol. IX/2436, Ver 7, Feb 23rd, 2021). This trial was registered at ClinicalTrials.gov (NCT04563546).

## Results

**Figure 1:**
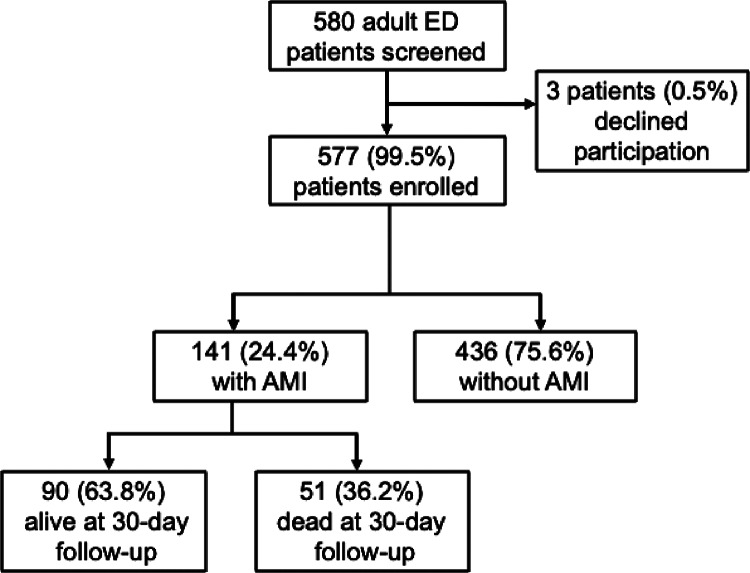
Flow chart of patient participants.

During the study period, 580 adult patients presented to the KCMC ED with chest pain or dyspnea and were eligible for enrollment. Of these, 577 (99.5%) consented to participate and were enrolled. Of these, 141 (24.4%) met the study definition for AMI. At the 30-day follow-up, 90 (63.8%) participants with AMI were still alive (Figure 1). During the study period, 22 physicians and 32 nurses worked in the KCMC ED.

### Fidelity

A designated physician and nurse champion were appointed for the first six months of the study period; the Head of the ED subsequently decided to appoint a new physician and nurse champion for the second six-month period to allow for broader staff engagement with the champion role. Champions remained engaged throughout the entire twelve-month study period, performing auditing on at least a monthly basis and distributing congratulatory certificates on a semi-annual basis. There was excellent fidelity to the pocket cards, with 100% of the total 22 physicians observed carrying their pocket cards with them to work on at least one occasion (Table 1). With regard to the online training module, a large majority of ED physicians (20 of 22, 90.9%) started the module, however, a lower proportion of ED nurses started the module (25 of 32, 78.1%). With regard to the triage card component of the intervention, 453 (78.5%) of the 577 patients with chest pain or dyspnea received the “AMI Suspect” triage card. Fidelity to the patient educational component, however, was significantly lower, with only 53 (38%) of the 141 patients with AMI receiving a pamphlet.

**Table 1:** Fidelity of implementation of the MIMIC intervention in the KCMC ED, 2023–2024.

FIDELITY MEASURE	DENOMINATOR	TOTAL N	OBSERVED N	%
Proportion of participants with chest pain/dyspnea correctly flagged the “AMI Suspect” triage card	Patient participants with AMI symptoms	577	453	78.5%
Proportion of physicians observed to have ever brought their pocket cards to work	ED physicians	22	22	100%
Proportion of ED staff starting the online training module	Total number of ED physicians and nurses	54	45	83%
Proportion of ED physicians starting the online training module	Total number of ED physicians	22	20	91%
Proportion of ED nurses starting the online training module	Total number of ED nurses	32	25	78%
Proportion of participants with AMI receiving the educational pamphlet in the ED	Total number of patient participants with confirmed AMI	141	53	38%

**Figure 2:**
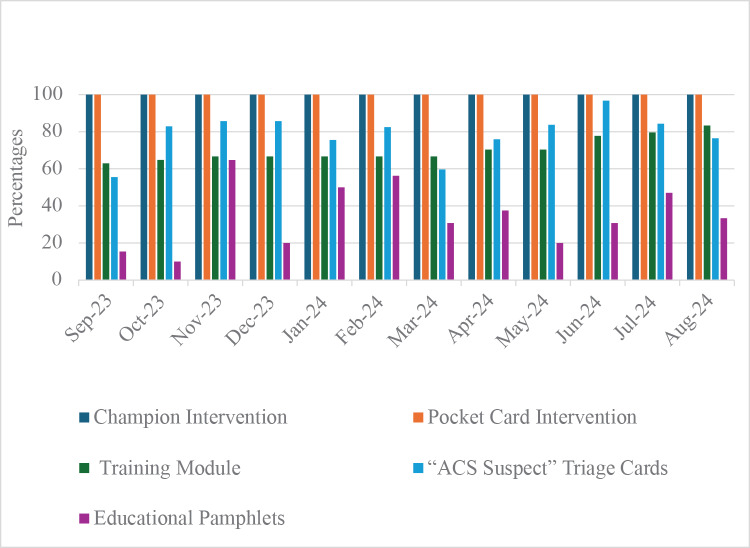
Monthly fidelity outcomes over the study period (September 2023–August 2024).

Monthly fidelity outcomes are presented in Figure 2. There was 100% fidelity to the champion intervention and the pocket card intervention over the twelve-month study period. Fidelity to the training module improved gradually over time; 63% (34 of 54) of staff started the training module in the first month, rising to 83% (45 of 54) of staff in the final month. Fidelity to “ACS Suspect” triage cards and educational pamphlets varied substantially across months.

### Penetration

Table 2 presents the penetration of the various MIMIC components among target audiences. The intervention component with the greatest penetration was pocket cards, with pocket cards being directly observed at work for 96.1% (1835/1910) of discrete physician-shifts. Penetration of the online training module was greater among physicians than nurses, with 20 (91%) of 22 physicians completing the module but only 21 (66%) of 32 nurses doing so. Penetration of the “AMI Suspect” triage cards among nurses working in triage was observed for only 67.2% (572/851) of discrete nurse triage-shifts. Of the 53 participants with AMI who received a pamphlet, 39 survived to thirty-day follow-up. Penetration of the educational pamphlets was low: only 22 (56%) of the 39 surviving AMI patients who received a copy reported reading the pamphlet. Among the 17 surviving patients who received the pamphlet but did not read it, 15 reported they were not aware they had received it, 1 reported they forgot to read it, and 1 reported they had lost it.

**Table 2:** Penetration-related findings.

PENETRATION MEASURE	DENOMINATOR	TOTAL N	OBSERVED N	%
Proportion of nurses working in triage directly observed to use the “AMI suspect” triage card	Discrete nurse triage-shift observations	851	572	67.2%
Proportion of physicians directly observed to have their pocket cards at work	Discrete physician-shift observations	1910	1835	96.1%
Proportion of ED providers completing the online training module	All ED staff	54	41	76%
Proportion of ED physicians completing the online training module	All ED physicians	22	20	91%
Proportion of ED nurses completing the online training module	All ED nurses	32	21	66%
Proportion of surviving participants with AMI who read the educational pamphlet within 30 days	AMI patients who received the educational pamphlet and survived to 30 days	39	22	56%

### Cost

The total cost of the MIMIC intervention over the one-year pilot period was USD 1,324.24 (Table 3). The champion stipends ($1044.50) accounted for most (79%) of the total cost of the intervention. A graduate research assistant spent approximately 8 hours building and editing the online module.

**Table 3:** Financial cost summary of MIMIC implementation for 12 months.

EXPENSE	TSH	USD
Champion stipends (One physician and one nurse for 12 mo.)	2,400,000	$1,044.50
Printing triage cards (50 printed and laminated)	62,500	$27.20
Printing pocket cards (140 printed and laminated)	145,000	$64.10
Printing certificates (4 printed)	8,000	$3.48
Printing educational pamphlets (280 printed)	425,000	$184.96
Building the online module (8 hours)	330,900	$144.00
Total cost of implementation:	3,371,400	$1,468.24

## Discussion

To our knowledge, this is the first study of an intervention with specific strategies aimed at improving the uptake of evidence-based AMI care in Sub-Saharan Africa. In this pilot trial of the MIMIC intervention in an ED in northern Tanzania for 577 enrolled patient participants presenting with AMI symptoms, we examined key implementation outcomes, including fidelity, penetration, and cost. While champions and physician pocket cards became part of standard practice, completing the online training modules, identifying patients with AMI symptoms and providing patient education was possible but less than optimal. This pilot trial will inform future scale-up efforts of the intervention in Tanzania.

Fidelity of intervention components was highly variable. Nurses used the AMI Suspect triage card for three of every four patients enrolled presenting with symptoms. Fidelity to the pocket card intervention and the training intervention (particularly among physicians) was much higher than fidelity to the educational pamphlet. Having less than half of patients with AMI receiving the pamphlet may reflect provider attitudes toward the level of priority for distributing educational materials compared with caring for critically ill patients. Some patients may have been considered too ill to receive them. Fidelity to this component may have been higher if the pamphlets were distributed in a less acute setting, such as the inpatient ward. Prior quantitative and qualitative studies have shown that there is a tremendous need for more AMI education among patients in Tanzania and that limited AMI knowledge among patients is a significant barrier to care [7, 8, 23, 24]. Notably, many studies of educational interventions for patients with AMI have not reported fidelity to the intervention, making it difficult to compare implementation outcomes with similar interventions in other settings [25, 26]. Thus, identifying an effective strategy for educating patients, and ensuring their awareness and understanding (penetration), is crucial prior to further scale-up of the intervention. Fidelity to the champions, pocket cards, and training module was sustained or improved over time following the implementation of MIMIC, but there were no clear time-related trends in fidelity to the triage cards or educational pamphlets. Further study will need to evaluate the long-term fidelity of these intervention components and normalization into routine care.

Penetration trended similarly to measures of fidelity, with penetration of the intervention components being generally higher among physicians than nurses or patients. Only two-thirds of ED nursing shifts used the “AMI Suspect” triage cards and two-thirds of nurses completed the online training module. The reasons for lower penetration of the intervention among nurses warrant further study, particularly since KCMC ED nurses co-designed the MIMIC intervention [13]. It is possible that implementation did not consider the potential impact of staffing shortages, as the triage area in the ED is occasionally staffed by nursing students or nurses from other departments. The nurse champion’s role in oversight could be revisited for future implementation. Similarly, the nurse champion could leverage different approaches to support nurse completion of online training, such as access to a computer and time to complete it. Content will also be re-reviewed to ensure both the roles of nurses and physicians are both adequately represented. Notably, our measure of penetration for the triage card component (proportion of nurse-shifts directly observed to be using the triage cards) was dependent on the volume of patients with chest pain during the shift, unlike our measures of penetration for the pocket card and online module components, which were independent of case volume. This difference may partially explain lower penetration of the triage card component, but it does not explain the difference in online module penetration between physicians and nurses. Among the AMI patients who received the educational pamphlet, a substantial proportion reported not being aware that they had received the pamphlet. This is a surprising finding that warrants further exploration. Distributing the pamphlets in a setting with less acuity, such as a follow-up clinic or inpatient ward, may improve penetration of this intervention.

The total annual cost of the MIMIC intervention was USD 1324, which is less than the cost of an ECG machine in Tanzania. Most of the cost of the MIMIC intervention was due to the champion stipends. Cost-effectiveness analyses will be needed to determine the costs associated with the effective delivery of care to justify future scale-up of the intervention. If the MIMIC intervention does increase the uptake of evidence-based AMI care and reduce mortality, it may be highly cost-effective. The cost-effectiveness of the intervention could be enhanced if non-monetary means of compensating champions are identified.

This study had several limitations. First, the proportion of patients reading the educational pamphlet was measured by patient self-report, which may have resulted in over-estimation of the penetration of this component due to social desirability bias. Second, as with any observational study, the Hawthorne effect may have influenced our results for physician and nurse behavior, resulting in over-estimation of fidelity and penetration of the intervention. Third, our measurement of intervention costs focused exclusively on monetary cost; other costs, such as the amount of time ED staff spent implementing the MIMIC intervention, were not included in this outcome.

In conclusion, in the first trial of a multicomponent intervention to improve the uptake of evidence-based AMI care in Tanzania, we found fidelity and penetration varied for different intervention components. Future studies are needed to further explore the barriers of co-designed components, and revisit strategies for patient education. Analysis of the effect of the MIMIC intervention on AMI care delivery and outcomes, and the cost-effectiveness of care are needed to inform future efforts to scale up MIMIC across Tanzania.

## Data Availability

All authors had access to the study data and had a role in writing the manuscript.
